# An M5Stamp Pico-Based IoT Soil Monitoring System for Soil Water–Salinity Diagnosis in a Coastal Reclaimed Pepper Greenhouse

**DOI:** 10.3390/s26113309

**Published:** 2026-05-22

**Authors:** Leon Nakayama, Ieyasu Tokumoto

**Affiliations:** Faculty of Agriculture, Saga University, 1 Honjo, Saga 840-8502, Japan

**Keywords:** pore-water electrical conductivity, osmotic potential, reclaimed polder

## Abstract

Coastal reclaimed polders with shallow saline groundwater support intensive greenhouse horticulture but require timely diagnosis of root-zone water and salinity conditions. This study developed a compact Internet-of-Things (IoT) monitoring system based on the M5Stamp Pico microcontroller to acquire SDI-12 soil-sensor data, buffer records locally, and transfer them to a low-cost cloud dashboard. Outside-greenhouse validation showed high operational reliability, with a missing observation rate of only 0.9%, and acceptable agreement with a reference TDR100 for both volumetric water content (θ) and bulk electrical conductivity (EC_b_). The system was then applied to ridge-position monitoring in a commercial pepper greenhouse on a coastal reclaimed polder. The ridge records captured depth-dependent infiltration and salinity redistribution under drip irrigation, together with contrasting responses between the cultivated layer and shallow groundwater. Potential-based interpretation indicated that the monitored ridge root zone was often not strongly limited by matric potential, whereas osmotic potential derived from pore-water salinity showed reduced water availability even when the soil remained relatively wet. These results demonstrate that continuous real-time monitoring at the ridge position can support diagnosis of root-zone stress and provide useful information for irrigation and fertigation management in salt-affected greenhouse soils.

## 1. Introduction

Coastal reclaimed polders support intensive greenhouse horticulture, but shallow saline groundwater keeps the root zone vulnerable to salt accumulation. In such environments, capillary rise, irrigation, and drainage jointly control whether salts are leached from or retained in the cultivated layer [1,2,3]. Because greenhouse covering and ridge–inter-row management modify surface wetting and evaporation, salinity risk can vary markedly within short distances and over short times [1,2,3]. Reliable crop production therefore requires monitoring methods that can resolve both water and salinity conditions in the root zone.

Electromagnetic methods such as time-domain reflectometry/transmissiometry (TDR/TDT) are attractive for this purpose because they allow repeated in situ measurements of bulk dielectric permittivity and bulk electrical conductivity, from which volumetric water content (θ) and salinity-related indicators can be derived [4,5,6]. However, performance in saline and clay-rich soils is not straightforward: sensor response depends on salinity level, water content, and calibration strategy, and systematic bias may appear if sensors are used without local validation [7,8]. For practical monitoring in reclaimed soils, sensor performance therefore needs to be verified under field conditions rather than assumed from factory specifications alone.

For agronomic interpretation, EC_b_ alone is not sufficient because plant water uptake is influenced by the salinity of the soil solution rather than bulk conductivity itself. Converting EC_b_ to pore-water electrical conductivity (EC_w_) and then to osmotic potential provides a more plant-relevant basis for diagnosing salinity-related restriction of water uptake [5,6,9]. This is especially important in saline reclaimed soils, where the root zone may appear wet from θ alone while still being physiologically unfavorable for crop growth.

At the same time, the data-acquisition system remains a major barrier to the routine use of soil water–salinity monitoring in agricultural fields. Conventional TDR/TDT monitoring often depends on proprietary loggers and communication hardware, which increases cost and reduces flexibility for distributed observations. Wireless sensor networks and low-cost platforms have been applied to soil monitoring in salt-affected or disaster-affected farmlands [10,11,12,13], but practical barriers still remain when such systems are extended to routine operation by farmers or small-scale field users.

Previous studies by our group and related work have demonstrated the value of real-time monitoring for salinity-affected farmlands [1,10,12]. Tokumoto et al. [10] showed that a field monitoring system enabled real-time observation of tsunami-affected farmlands, and Tokumoto et al. [1] further demonstrated the usefulness of multiple-point WSNS observations in a heterogeneous reclaimed greenhouse after the Kumamoto Earthquake. However, in our previous development and field operation of such systems, practical limitations became apparent for routine on-farm use. In particular, server maintenance and system management became burdensome as the number of measurement points increased, and Arduino-based logging systems required program modifications depending on the connected SDI-12 sensors. These practical constraints reduced the ease of long-term operation in commercial settings. Therefore, beyond sensing accuracy alone, practical operability, ease of maintenance, and low-cost data access are critical requirements if such monitoring systems are to be used directly by farmers.

To address these practical barriers, the novelty of the present study does not lie in a new dielectric inversion algorithm, salinity model, or automated control logic. Rather, it lies in the practical integration of professional SDI-12 soil sensors, a compact M5Stamp Pico microcontroller, local microSD buffering against communication loss, and a low-cost cloud workflow based on automatic writing to and retrieval from Google Sheets for remote visualization and data access. This design was motivated by the need for a monitoring platform that reduces operational and maintenance burdens while remaining simple enough for practical use by farmers and compatible with professional soil sensors used for root-zone water and salinity diagnosis. Recent studies have further coupled sensing with irrigation scheduling or control frameworks, including automated water-use control and soil-moisture-, simulation-, or weather-based irrigation scheduling [14,15,16]. These studies are relevant because they show how sensor information can be translated into management actions. In contrast, the present study addresses the earlier step of field-operable acquisition, calibration, and interpretation of root-zone water–salinity data in a saline reclaimed greenhouse soil; it does not claim to have implemented irrigation-trigger logic or closed-loop irrigation control.

In this study, a compact IoT soil-monitoring system based on the M5Stamp Pico microcontroller was developed and evaluated for a commercial pepper greenhouse on a coastal reclaimed polder. The study aimed to develop a practical SDI-12 wireless node for continuous soil sensing and to verify its measurement performance and data continuity under outside-greenhouse field conditions. The system was then applied to ridge-position monitoring in a commercial greenhouse to examine root-zone water–salinity conditions and assess the practical operability of the proposed Google-Sheets-based workflow for on-farm use.

## 2. Materials and Methods

### 2.1. Wireless Sensor Node Based on M5Stamp Pico

#### 2.1.1. Embedded Hardware and SDI-12 Interface

**Figure 1:**
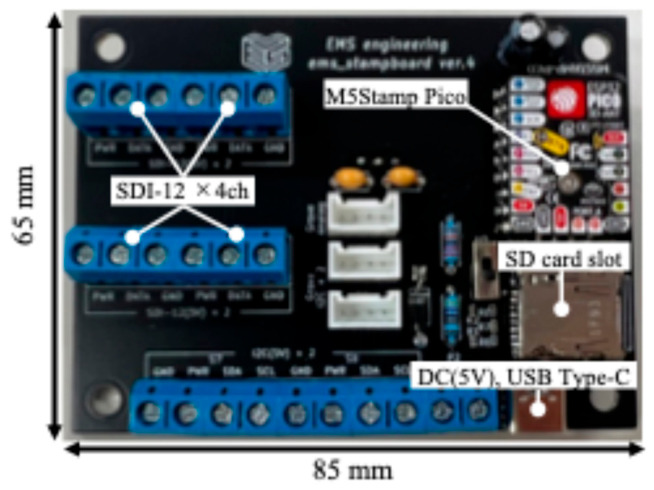
Photograph of the custom Stamp Board used as the field monitoring node. The board includes the M5Stamp Pico module, SDI-12 terminal blocks, a 5 V power input, and a microSD card slot.

A compact wireless sensor node was developed around the M5Stamp Pico microcontroller module (M5Stack Co., Ltd., Shenzhen, China), which incorporates an ESP32 system-on-chip with integrated Wi-Fi communication [13]. The M5Stamp Pico was mounted on a custom Stamp Board developed for field soil monitoring (Figure 1). The board included a four-channel SDI-12 terminal block, a 5 V power conditioning circuit, and a microSD card slot for optional local data storage. Its dimensions were approximately 65 mm × 85 mm, allowing installation inside a compact waterproof enclosure. The node was programmed using the Arduino framework (Arduino IDE, Arduino, https://www.arduino.cc/) and powered from an AC adapter (5 V) connected to a power strip in the greenhouse.

**Figure 2:**
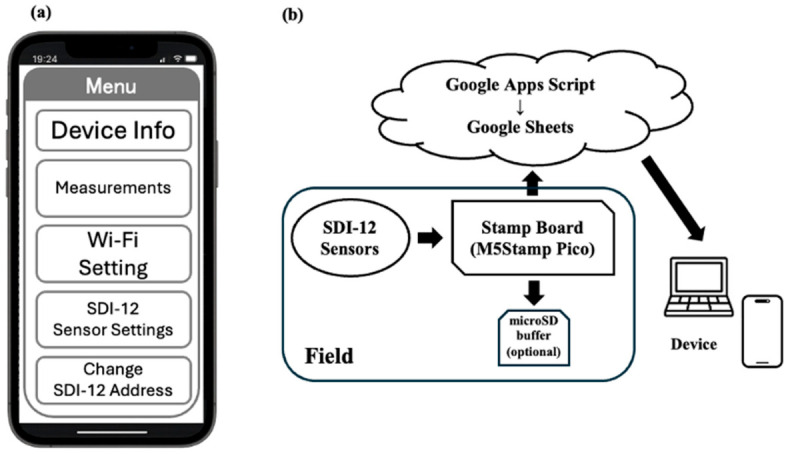
(**a**) Smartphone application developed for field setup and checking of the SDI-12 monitoring node; (**b**) overview of the wireless node hardware configuration and end-to-end data flow from SDI-12 sensing to the cloud dashboard.

To simplify field setup, we developed a smartphone application for SDI-12 sensors (Figure 2a). The application allowed users to check device information, confirm sensor measurements, set Wi-Fi parameters, configure SDI-12 sensor settings, and change sensor addresses. Sensors assigned to addresses 0–9 could be queried without modifying the main logging program. This function reduced the need for sensor-specific code changes and made it easier to confirm sensor operation during installation, replacement, and routine field checks.

For soil measurements, SDI-12-compatible time-domain reflectometry (TDR) sensors, such as the Acclima TDR315H (Acclima, Inc., Meridian, ID, USA), were connected to the Stamp Board through the SDI-12 terminal block. Each sensor returned bulk dielectric permittivity (ε_b_), bulk electrical conductivity (EC_b_), and sensor-internal temperature through the manufacturer’s SDI-12 protocol. This design enabled professional SDI-12 soil sensors to be integrated into a compact low-cost monitoring node. Figure 2b shows the overall configuration from SDI-12 sensing to the field node, optional local buffering, cloud transfer, and remote device access; the cloud logging and visualization workflow is described in the next subsection.

#### 2.1.2. Cloud Data Logging and Visualization (Google Sheets with Google Apps Script)

A cloud-based logging and visualization workflow was built with Google Sheets and Google Apps Script (GAS) (Google LLC, Mountain View, CA, USA; https://www.google.com/sheets/about/ and https://developers.google.com/apps-script, accessed on 19 May 2026). Google Sheets served as the receiving database and dashboard because it could store multichannel records and display time-series data quickly.

A dedicated Google Sheets file on Google Drive received URL-encoded records through a GAS web application. Each record was appended with its timestamp and sensor identifier. The spreadsheet was then used to inspect the time series and download archived data for analysis. This workflow allowed θ and EC_b_ to be visualized automatically during routine greenhouse operation.

### 2.2. Study Site and Greenhouse Management

**Figure 3:**
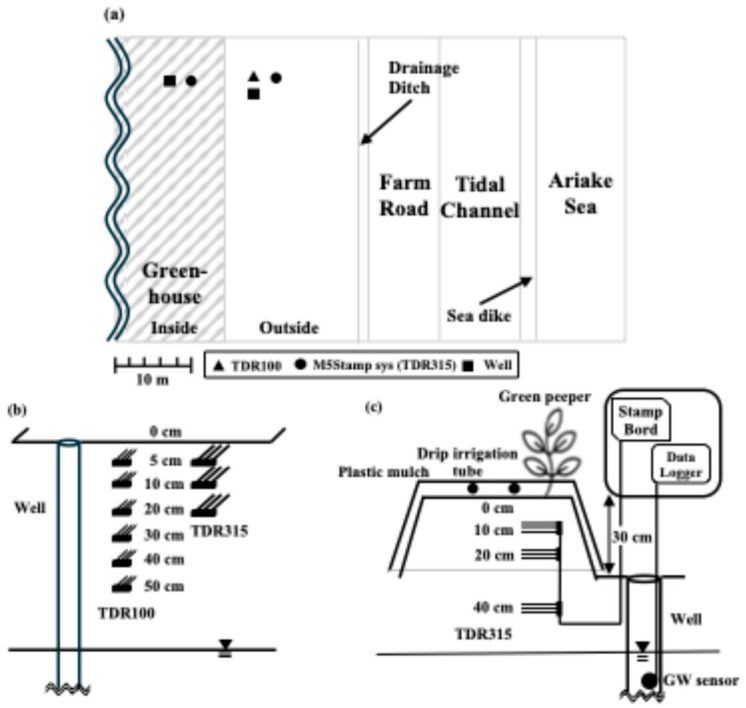
Study site and monitoring configuration. (**a**) Location of the pepper greenhouse on the Yokoshima reclaimed polder and the positions of the outside validation plot and observation well. (**b**) Outside-greenhouse validation setup showing the installation depths of the IoT-connected TDR315 sensors and the reference TDR100 probes. (**c**) In-greenhouse cross-sectional schematic of the cultivated ridge, adjacent well, and groundwater sensor under plastic mulch and drip irrigation. GWL denotes groundwater level, and GW denotes groundwater.

The study was conducted in a commercial pepper greenhouse located on a coastal reclaimed polder in Kumamoto Prefecture, Japan (Figure 3a). The polder is characterized by shallow saline groundwater and a layered soil profile, with a sandy surface layer overlying clayey subsoil. The groundwater table is typically within approximately 1.0 m of the ground surface (Figure 3b), and groundwater electrical conductivity is elevated due to residual sea-salt ions associated with the reclamation history [1].

Peppers were cultivated on raised ridges covered with black polyethylene mulch. Drip irrigation was supplied beneath the mulch. Subsurface drainage pipes installed beneath the greenhouse were connected to an outlet that could be opened or closed to help manage groundwater level in the greenhouse. The greenhouse layout is shown in Figure 3a, and the cultivated ridge and monitoring configuration are shown in Figure 3c.

### 2.3. Outside-Greenhouse Sensor Performance Assessment

Outside-greenhouse measurements were conducted in an uncultivated area adjacent to the commercial greenhouse. The M5Stamp Pico-based node was operated with SDI-12 time-domain sensors under ambient meteorological conditions. In parallel, a conventional TDR system (TDR100; Campbell Scientific Inc., Logan, UT, USA) was used as a reference for comparison at the same site.

#### 2.3.1. Instrumentation and Sensor Installation Outside the Greenhouse

SDI-12 time-domain sensors connected to the wireless node were installed in the upper soil layer at nominal depths of 0.05, 0.10, and 0.20 m (measured from the soil surface) (Figure 3b). Sensors were inserted horizontally from the side wall of a shallow pit to minimize disturbance around the sensing rods; the pit was then backfilled and gently compacted.

For reference measurements, the TDR100 system was installed in close proximity to the IoT-connected sensors. At the study site, the TDR100 had already been used for medium-term monitoring, and θ derived from the TDR100 was treated as the reference because its interpretation was based on field calibration for the local soil conditions. The present comparison was therefore intended to evaluate the performance of the IoT-connected TDR315 against an established TDR-based reference under the same field environment. Before the present comparison, the soil profile for TDR100 monitoring had already been instrumented with TDR probes (CS614, Campbell Scientific Inc., USA) at depths of 0.05, 0.10, 0.20, 0.30, 0.40, and 0.50 m. Among these measurements, the records at 0.05, 0.10, and 0.20 m were used as reference data for comparison with the IoT sensors, whereas the deeper probes provided information on water content in the lower soil profile. The installation distance between the TDR probes and the IoT probes was kept as small as practicable while avoiding mutual interference between probes.

To provide an independent power supply for the entire monitoring system, all sensor nodes and associated communication components were operated using a 20 W solar panel and a 20 Ah lead–acid battery. Data acquired by each sensor node at 30 min intervals were transmitted to a Google Spreadsheet through LTE communication via a mobile router (FS030WMB1, Fujisoft Inc., Kanagawa, Japan). By executing the same communication routine on each M5Stack-based board, records from multiple nodes could be integrated into a single Google Spreadsheet dashboard (Figure 2) for visualization and remote inspection on smartphones or computers.

#### 2.3.2. Measurement Operation and Data Handling

Outside-greenhouse validation was conducted from 14 January to 15 February 2025. The wireless node queried the SDI-12 sensors at 30 min intervals and recorded bulk dielectric permittivity (ε_b_), bulk electrical conductivity (EC_b_), and sensor-internal temperature together with timestamps. The TDR100 reference system was recorded every 3 h, whereas meteorological measurements were recorded every 10 min.

Agreement between the IoT-connected sensors and the reference TDR100 was evaluated at each depth using root mean square error (RMSE), Pearson correlation, and mean bias. Paired observations were formed without interpolation by rounding timestamps from both series to the nearest 30 min and retaining only coincident records.

### 2.4. In-Greenhouse Monitoring at the Ridge Position Under Pepper Cultivation

Following the outside-greenhouse validation, the same monitoring system was operated in the commercial pepper greenhouse on the reclaimed polder site. For the analyses presented in this paper, ridge-position measurements directly relevant to crop root-zone diagnosis were examined.

#### 2.4.1. Sensor Installation and Monitoring Configuration at the Ridge Position

Peppers were cultivated on raised ridges covered with black polyethylene mulch, and irrigation water and fertilizer were supplied by drip irrigation beneath the mulch. Subsurface drainage pipes installed beneath the greenhouse were connected to an outlet whose opening was adjusted by the farmer (Figure 3c).

SDI-12 time-domain sensors were installed at nominal depths of 0.10, 0.20, and 0.40 m in one representative cultivated ridge. Probes were inserted horizontally from the side wall of a small pit, followed by careful backfilling and compaction to restore soil contact around the rods. Although additional observations were made during field operation, the present paper focuses on ridge-position records to evaluate the monitoring system and diagnose root-zone conditions relevant to crop growth.

#### 2.4.2. Monitoring Period and Groundwater Measurements

For the analyses in this paper, the interval from 15 February 2025 to 7 March 2025 was selected because soil and groundwater records overlapped continuously and covered the period used for the greenhouse monitoring analysis shown below. Because the greenhouse was covered, precipitation did not enter the monitored ridge directly. Groundwater level (GWL) and groundwater electrical conductivity (ECGW) were measured with a groundwater sensor (Hydros 21, Meter Group, Inc., Pullman, WA, USA) at 10 min intervals in an observation well near the greenhouse. These groundwater data were obtained independently of the Stamp Board system and were used only to aid interpretation of the ridge measurements.

### 2.5. Conversion of Sensor Outputs to θ, EC_w_, and ψ_m_

Raw SDI-12 outputs from the time-domain sensors—ε_b_, EC_b_, and sensor-internal temperature—were post-processed to derive θ, pore-water electrical conductivity (EC_w_), and matric potential (ψ_m_). Volumetric water content was calculated from ε_b_ using a site-specific calibration, EC_w_ was estimated from ε_b_ and EC_b_ with a Hilhorst-type relationship, and ψ_m_ was obtained by mapping θ to pressure head through measured soil water retention curves. We chose established, physically interpretable relationships so that the processing framework remained comparable with previous reclaimed soil studies [1,6,10] and suitable for field diagnosis.

#### 2.5.1. Estimation of θ and EC_w_

Volumetric water content was estimated from ε_b_ using a site-specific calibration developed for reclaimed soils. A second-order polynomial relationship was adopted:

θ = 0.0604 + 0.0204·ε_b_ − 0.00020·ε_b_^2^, (1)

where θ (m^3^ m^−3^) is the volumetric water content and ε_b_ (–) is the bulk dielectric permittivity. The coefficients were determined from laboratory calibration using soil samples collected from the study polder.

Pore-water electrical conductivity was estimated from bulk measurements using a Hilhorst-type relationship:

(2) $${EC}_{w}=\frac{\varepsilon_{w}\cdot {EC}_{b}}{\varepsilon_{b}-\varepsilon_{0}},$$

where ε_w_ is the dielectric permittivity of free water (ε_w_ = 78.5 at 25 °C), and ε_0_ is an offset permittivity reflecting soil-specific conduction in the solid phase. In this study, ε_0_ was set to 5.51, 6.08, and 6.70 for sensors at 0.10, 0.20, and 0.40 m, respectively, based on saturated-paste calibration; temperature effects on ε_w_ were neglected by standardizing to 25 °C.

The θ calibration and the ε_0_ values used for EC_w_ estimation were determined from local soil samples, following the approach used in previous soil monitoring studies [1]. The ε_b_–θ relationship was obtained by laboratory calibration of the TDR sensor, and ε_0_ was checked using saturated-paste measurements and a Rhoades-type bulk–solution conductivity relationship. For routine field monitoring, the Hilhorst-type equation was adopted because it estimates EC_w_ directly from ε_b_ and EC_b_, which are recorded by the SDI-12 sensor. This simplicity made the calculation suitable for implementation in the Google-Sheets-based workflow for near-real-time monitoring by farmers. These calibration parameters are soil-specific and should be redetermined before the workflow is applied to another site.

#### 2.5.2. Soil Water Retention Curve Measurement and Estimation of ψ_m_

Matric potential (ψ_m_) was estimated from dielectric-derived θ using soil water retention characteristics measured for the cultivated ridge at depths corresponding to 0.10, 0.20, and 0.40 m. Soil water retention data were obtained by combining (i) a suction method for the wet range and (ii) a dewpoint potentiometer (WP4, METER Group, Inc., Pullman, WA, USA) for the dry range.

For the wet range, pressure head was imposed stepwise down to h = −120 cm; after equilibration at each step, gravimetric water content was measured and converted to θ using sample bulk density. For the dry range (h < −120 cm), water potential measured with WP4 (ψ, MPa) was converted to pressure head (h, cm) as:

(3) $$h=\frac{\psi \times {\;10}^{6}\;\times \;100}{\rho_{w}g},$$

The combined suction–WP4 datasets were fitted with the Durner two-modal (two-stage drainage) soil water retention model:

(4) $$\theta \left(h\right)=\theta_{r}+\left(\theta_{s}-\theta_{r}\right)\cdot \left[\frac{1-w_{2}}{{\left(1+{\alpha_{1}\left|h\right|}^{n_{1}}\right)}^{m_{1}}}+\frac{w_{2}}{{\left(1+{\alpha_{2}\left|h\right|}^{n_{2}}\right)}^{m_{2}}}\right],\;\mathrm{with}\;m_{i}\;=\;1\;-\;1/n_{i}\;(i\;=\;1,\;2)$$

where θ_s_ and θ_r_ are the saturated and residual water contents, 0 < w_2_ < 1 is the weighting factor, and α_i_ and n_i_ are fitting parameters for each pore domain. Table 1 lists the fitted parameters used to derive ψ_m_ from θ in this study.

For each 30 min observation, dielectric-derived θ was mapped to the corresponding pressure head by numerically inverting the fitted retention function θ(h). Matric head was expressed as ψ_m_ = −h (cm), following the conventional sign convention in soil physics. The resulting ψ_m_ series were used for subsequent interpretation of soil water status.

## 3. Results and Discussion

### 3.1. Monitoring System Verification and Operational Reliability

#### 3.1.1. IoT-Connected TDR Measurements in the Upper Soil Layer

**Figure 4:**
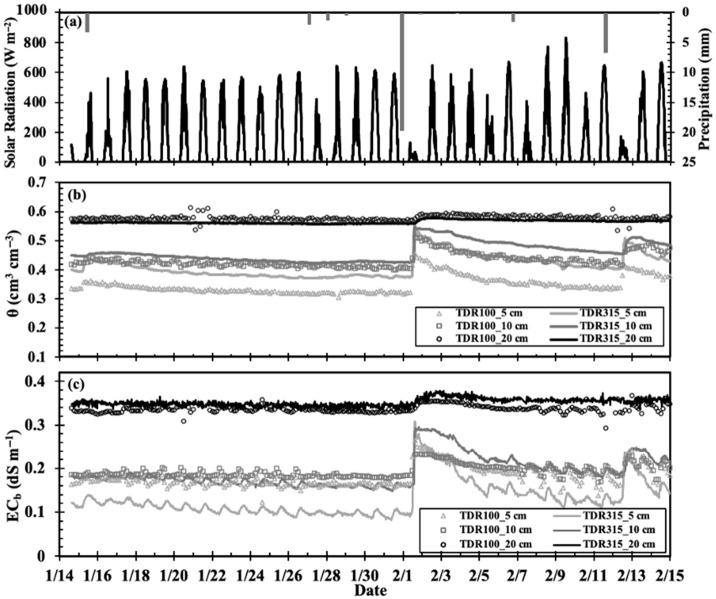
Outside-greenhouse validation: (**a**) meteorological forcing represented by solar radiation and precipitation; (**b**) comparison of volumetric water content (θ) measured by the reference TDR100 and the IoT-connected TDR315 at 0.05, 0.10, and 0.20 m; and (**c**) comparison of bulk electrical conductivity (EC_b_) at the corresponding depths.

During the outside-greenhouse evaluation period (14 January–15 February 2025), daily peak solar radiation ranged from 134.3 to 830.4 W m^−2^ (Figure 4a). A precipitation event totaling 19.7 mm occurred on 1 February 2025. Daily integrated solar radiation, calculated from the 10 min meteorological records, averaged 11.3 MJ m^−2^ d^−1^ on rain-free days. These conditions provided a suitable range of atmospheric forcing for evaluating the field performance of the IoT-connected TDR315 sensor under outside conditions.

Across the validation period, the IoT-connected TDR315 reproduced the θ changes measured by the reference TDR100 at 0.05–0.20 m (Figure 4b). Agreement statistics showed clear depth dependence (Table 2). At 0.05 m, Pearson’s r was highest, but RMSE and mean bias were also largest, suggesting greater sensitivity to near-surface heterogeneity such as preferential infiltration, evaporation gradients, and differences in sampling volume between adjacent probes. At 0.10 m, RMSE and bias were smaller while correlation remained high, indicating the most balanced agreement. At 0.20 m, RMSE was smallest and the two time series agreed visually, but Pearson’s r declined because θ varied over a narrower range. Thus, the lower correlation at 0.20 m reflects limited temporal variation rather than poorer agreement. This pattern is consistent with reclaimed-soil studies in which shallow layers responded more strongly to surface forcing than deeper layers influenced by groundwater [1,6].

Under the unfertilized outside conditions, EC_b_ remained low throughout the profile (Figure 4c). Across 0.05–0.20 m, EC_b_ ranged from 0.051 to 0.273 dS m^−1^. RMSE against the TDR100 reference was 0.036, 0.017, and 0.030 dS m^−1^ at 0.05, 0.10, and 0.20 m, respectively. These results indicate that the IoT-connected TDR315 measured low background EC_b_ with sufficient accuracy across the monitored depths.

The continuity of the outside record further demonstrated operational reliability. During 14 January–15 February 2025, only 14 of 1554 expected 30 min observations were missing, equivalent to a missing-data rate of 0.9% and a data availability of 99.1%. Because the wireless node buffered records locally during temporary Wi-Fi loss, this result indicates robust sensing, logging, and data transfer under field conditions. Such continuity is important in greenhouse monitoring, where interpretation of water and solute dynamics depends on uninterrupted records.

The present outside-greenhouse evaluation was designed as a field verification against an established reference system rather than as a multi-platform benchmark. Accordingly, the emphasis was placed on agreement metrics, continuity of logging, and depth-dependent behavior under field conditions. Broader generalization across sites, seasons, or alternative low-cost platforms will require additional comparative datasets.

#### 3.1.2. Vertical Soil Water–Salinity Profiles in the Uncultivated Outside Plot

**Figure 5:**
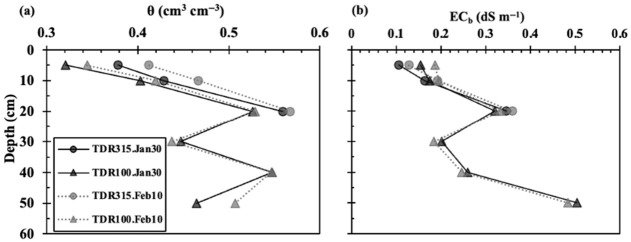
Vertical profiles in the uncultivated outside plot before and after the rainfall event on 1 February 2025: (**a**) volumetric water content (θ); (**b**) bulk electrical conductivity (EC_b_).

To characterize the background hydrological and salinity conditions outside the greenhouse, vertical profiles measured below 0.20 m were examined before and after the rainfall event on 1 February 2025 (Figure 5). These profiles clarify how water and dis-solved salts were distributed in the non-cultivated outside soil.

The θ profile showed a clear contrast between the surface and lower layers (Figure 5a). In the surface layer, water content changed more noticeably over the 10-day interval, indicating that rainfall-derived water was temporarily stored near the soil surface and then decreased again, likely because of subsequent evaporation and redistribution. In contrast, the lower layer maintained relatively high θ with smaller temporal variation. This pattern suggests that the deeper profile was less affected by short-term atmospheric forcing and remained under persistent influence of shallow groundwater.

The EC_b_ profile showed that conductivity was consistently higher in the lower part of the soil profile than in the upper layer (Figure 5b). Although the outside plot remained overall low in salinity, the deeper layer retained larger EC_b_ values throughout the observation period, indicating greater salt accumulation below the surface soil. A similar tendency has been reported for the same reclaimed polder, where elevated salinity in the lower profile was associated with the influence of shallow saline groundwater [1].

### 3.2. Ridge-Position Soil Moisture and Salinity Dynamics Under Greenhouse Cultivation

**Figure 6:**
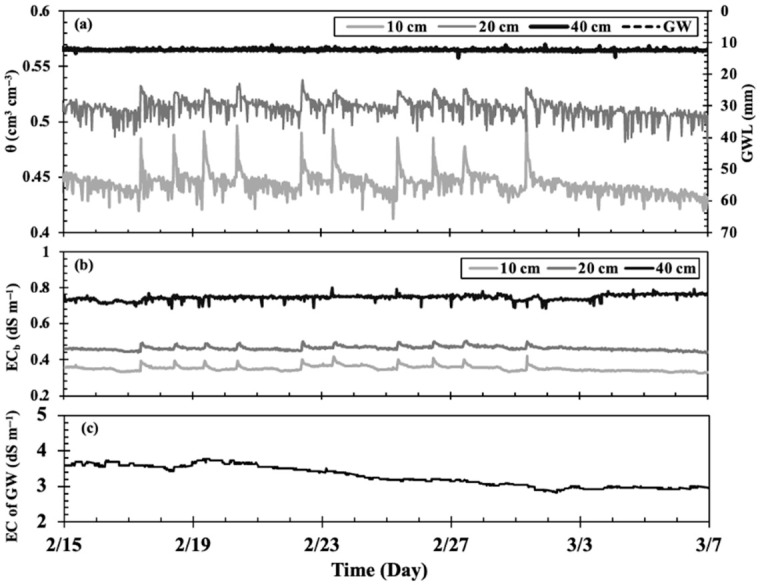
Time series of ridge-position soil water and salinity conditions during pepper cultivation (15 February–7 March 2025): (**a**) volumetric water content (θ) at 0.10, 0.20, and 0.40 m together with groundwater level (GWL, right axis); (**b**) bulk electrical conductivity (EC_b_) at the same depths; and (**c**) groundwater electrical conductivity (ECGW) measured in an adjacent observation well. The θ and EC_b_ records were acquired by the proposed IoT monitoring system, whereas the groundwater records were measured independently and are shown to support interpretation of the deeper soil response.

Figure 6 highlights one practical advantage of the proposed system. The Stamp Board continuously acquired ridge-position soil data from the SDI-12 sensors and automatically transferred θ and EC_b_ to Google Sheets, where the time series were generated with Google Apps Script. This workflow enabled near-real-time visualization of root-zone water and salinity conditions during greenhouse cultivation. In Figure 6, the soil records from the developed system are shown together with independently measured groundwater data. GWL is overlaid on the θ panel to aid interpretation of the deep response, whereas ECGW is plotted separately.

The continuous ridge record from 15 February to 7 March showed clear depth-dependent differences in soil water dynamics under drip irrigation (Figure 6a). Missing observations were negligible, confirming stable operation of the IoT system under cultivation conditions. At 0.10 m, θ showed repeated short-term increases followed by gradual declines. The response at 0.20 m was smaller and smoother. By contrast, θ at 0.40 m remained high and varied little, while the overlaid GWL record indicated persistently shallow groundwater. Thus, irrigation signals were strongest in the shallow cultivated layer, whereas the deeper layer was buffered by the groundwater environment.

The EC_b_ record also showed a clear depth dependence (Figure 6b). EC_b_ in the cultivated layer varied on a shorter time scale than θ, with clearer fluctuations at 0.10 and 0.20 m, whereas the deeper layer remained consistently higher throughout the monitoring period. Throughout the monitoring period shown in Figure 6, EC_b_ stayed below 1 dS m^−1^ at all depths, indicating a comparatively low bulk-salinity range in the monitored ridge. When viewed together with the independently measured ECGW record (Figure 6c), these plots allowed the salinity status of the root zone to be checked continuously under practical greenhouse operation. Compared with the severe post-earthquake salinity reported by Tokumoto et al. [1], in which drip irrigation alone did not control salinity, the present ridge remained much less saline. The workflow therefore provides a practical way to visualize whether ridge root-zone water and salinity conditions remain within a relatively favorable range for cultivation.

However, θ and EC_b_ alone do not fully describe plant-available water conditions in the root zone. Soil bulk density and water retention can differ with tillage and soil structure, so bulk measurements alone are not sufficient. For this reason, the monitored data were further converted to EC_w_, ψ_m_, and ψ_o_ to evaluate depth-dependent variation in root-zone water availability from a more plant-relevant perspective.

### 3.3. Potential-Based Diagnosis of Root-Zone Water and Salinity Conditions

The ridge measurements showed substantial depth and time dependence. To interpret these patterns in plant-relevant terms, the monitored data were further converted into matric potential (ψ_m_), pore-water electrical conductivity (EC_w_), osmotic potential (ψ_o_), and total potential (ψ_t_).

#### 3.3.1. Temporal Changes in Matric Potential, Pore-Water Electrical Conductivity, and Osmotic Potential

**Figure 7:**
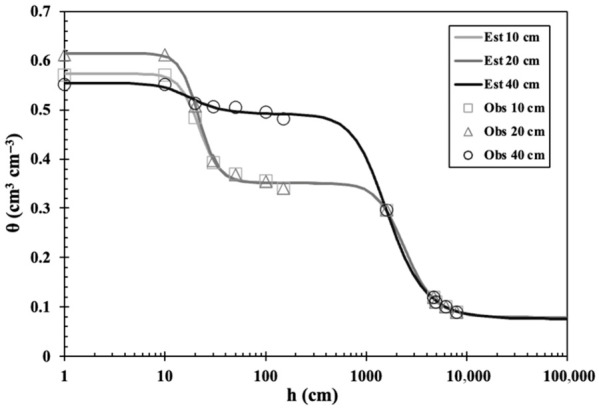
Soil water retention curves for the cultivated ridge at 0.10, 0.20, and 0.40 m and Durner model fits used to derive matric potential (ψ_m_) from θ.

Soil water retention curves measured for the cultivated ridge were used to convert θ to matric potential (ψ_m_) (Figure 7). The fitted curves showed clear depth dependence and strong nonlinearity between θ and h, indicating that similar θ values do not imply similar hydraulic conditions at all depths. To evaluate salinity-related restriction on water availability, EC_w_ and ψ_o_ were also derived from the monitored data.

**Figure 8:**
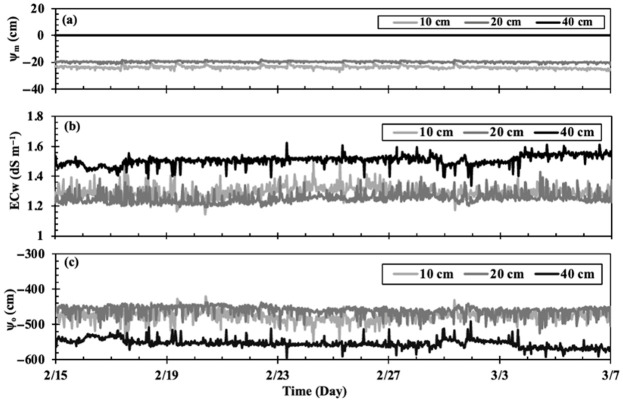
Temporal changes in (**a**) matric potential (ψ_m_), (**b**) pore-water electrical conductivity (EC_w_), and (**c**) osmotic potential (ψ_o_) at depths of 0.10, 0.20, and 0.40 m in the cultivated ridge during 15 February–7 March 2025.

Figure 8 shows the temporal changes in ψ_m_, EC_w_, and ψ_o_ at the ridge position during greenhouse cultivation. The ψ_m_ record stayed within a relatively narrow range throughout the monitoring period (Figure 8a), indicating that strong matric limitation did not develop in the monitored ridge during this interval. Among the three depths, ψ_m_ at 0.10 m was slightly more negative than at 0.20 and 0.40 m, whereas the 0.40 m layer remained relatively stable, consistent with buffering by the shallow groundwater table. Thus, hydraulic differences were modest compared with the salinity-related differences discussed below.

In contrast, EC_w_ showed a clearer and more persistent depth dependence (Figure 8b). The 0.40 m layer maintained the highest EC_w_ values throughout the period, the 0.20 m layer was intermediate, and the 0.10 m layer remained lowest. Short-term fluctuations were seen mainly in the upper ridge, suggesting repeated dilution and reconcentration of salts after irrigation and subsequent redistribution of soil water. Because ψ_o_ was calculated from EC_w_, its temporal pattern closely followed the salinity dynamics (Figure 8c). The 0.40 m layer consistently showed the most negative ψ_o_, whereas the 0.10 and 0.20 m layers remained less negative. Thus, salinity-related restriction on plant water uptake was strongest in the deeper layer, even though its matric condition remained relatively stable.

#### 3.3.2. Vertical Distribution of Total Potential and Implications for Root Uptake

To integrate matric and osmotic effects on root-zone water availability, total potential was calculated as:

(5) $$\psi_{t}=\psi_{m}+\psi_{o},$$

where ψ_t_ is the total potential. Because both ψ_m_ and ψ_o_ are negative in the present convention, more negative ψt values indicate less favorable conditions for root water uptake.

**Figure 9:**
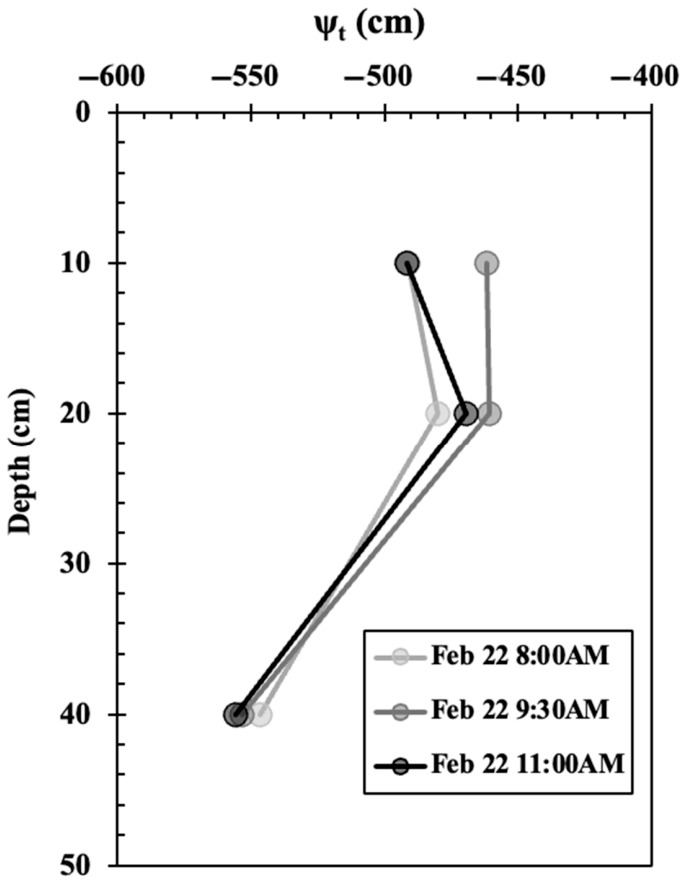
Vertical distribution of total potential (ψ_t_ = ψ_m_ + ψ_o_) in the cultivated ridge at 08:00, 09:30, and 11:00 on 22 February 2025, corresponding to before, during, and after drip irrigation over a 3 h period. More negative values indicate less favorable conditions for root water uptake.

Figure 9 shows an example of the vertical distribution of ψ_t_ at three times on 22 February, illustrating the change in total potential over the 3 h period before, during, and after drip irrigation. The profiles consistently separated the 0.40 m layer from the upper ridge: ψ_t_ at 0.40 m was markedly more negative than at 0.10 and 0.20 m at all three times. In contrast, the 0.10 and 0.20 m layers remained within a similar and more favorable range, although their order changed slightly with time. These profiles indicate that the upper to middle ridge provided more favorable combined hydraulic and osmotic conditions for root water uptake, whereas the deeper layer was relatively disadvantageous because of persistently strong osmotic effects.

This result is important for root-zone diagnosis in reclaimed greenhouse soils. A deeper layer may appear hydraulically stable because of groundwater influence, yet it may still be physiologically restrictive because of osmotic stress. By contrast, the upper and middle ridge may maintain more favorable total potential even when their short-term hydraulic responses differ. The combined use of ψ_m_, EC_w_, ψ_o_, and ψ_t_ therefore provides a more informative basis for evaluating root-zone conditions than θ or EC_b_ alone.

From a practical viewpoint, this potential-based interpretation may help farmers and advisers identify which soil layer is relatively favorable or unfavorable for root water uptake under ongoing fertigation. Although the present study did not implement an automated control function, the results suggest that real-time visualization of osmotic and total potential could provide a useful basis for future fertigation-support systems in salt-affected greenhouse soils.

The present interpretation should be viewed in light of several limitations. The monitoring period was short and restricted to a single commercial greenhouse on one reclaimed polder. Thus, long-term robustness and performance under other soils, salinity levels, and management conditions remain to be tested. In addition, the conversion of sensor outputs to θ, EC_w_, and ψ_m_ relied on site-specific calibration and local soil hydraulic characterization; therefore, these relationships should not be transferred directly to other soils without recalibration. Even so, the present results show that real-time visualization of ψ_m_, ψ_o_, and ψ_t_ can provide a practical basis for diagnosing root-zone conditions in saline greenhouse soils. Future work should test the system across sites and seasons and explore how these potential-based indicators can be incorporated into practical fertigation-support frameworks.

## 4. Conclusions

The proposed IoT-connected monitoring system enabled continuous real-time observation of ridge-position soil water and salinity conditions in reclaimed greenhouse soil under drip irrigation. The measurements showed that shallow and deeper parts of the cultivated ridge responded differently to water supply and that the ridge soil and shallow groundwater changed on different time scales. Potential-based interpretation further showed that matric potential was not strongly limiting during the analyzed interval, whereas osmotic effects made total potential substantially less favorable in the deeper layer than in the upper to middle ridge. These results demonstrate the practical value of the proposed system for ridge-scale diagnosis of root-zone conditions in salt-affected greenhouse soils.

## Figures and Tables

**Table 1:** Fitted parameters of the Durner model.

Depth (m)	θ_r_	θ_s_	α_1_	n_1_	w_2_	α_2_	n_2_
0.10	0.079	0.573	0.049	4.774	0.552	0.00048	3.397
0.20	0.079	0.615	0.049	5.087	0.509	0.00048	3.398
0.40	0.076	0.555	0.066	3.111	0.868	0.00076	2.831

**Table 2:** Agreement between IoT-connected TDR315 and reference TDR100 for volumetric water content (θ) during the outside-greenhouse validation period (14 January–15 February 2025), computed from paired observations at identical 30 min rounded timestamps aligned to the 3 h TDR100 schedule.

Depth (m)	*n* (Pairs)	RMSE (m^3^ m^−3^)	Pearson r	Mean Bias (IoT−TDR100)
0.05	255	0.066	0.952	0.065
0.10	257	0.031	0.905	0.027
0.20	255	0.015	0.463	−0.013

## Data Availability

The data presented in this study are available from the corresponding author upon reasonable request. The data is not publicly available because they include field records from a commercial greenhouse.
